# The influences of acculturation strategies on physician trust among internal migrants in Shanghai, China: a cross-sectional study in 2021

**DOI:** 10.3389/fpubh.2025.1506520

**Published:** 2025-04-23

**Authors:** Enhong Dong, Yue Yan, Sheng Ji, Jiabin Li, Tingting Wang, Jiahua Shi, Ting Xu, Weimin Gao, Yuping Liu, Shixiang Zhang

**Affiliations:** ^1^School of Nursing and Health Management, Shanghai University of Medicine & Health Sciences, Shanghai, China; ^2^Institute of Healthy Yangtze River Delta, Shanghai Jiao Tong University, Shanghai, China; ^3^School of Medicine, Tsinghua University, Beijing, China; ^4^HuangPu District Health Promotion Center, Shanghai, China; ^5^School of Nursing, Kunming Medical University, Kunming, China; ^6^School of Humanities and Management, Kunming Medical University, Kunming, China

**Keywords:** acculturation strategy, physician trust, internal migrant, megacity, China

## Abstract

**Purpose:**

This study aimed to clarify patterns of Berry’s acculturation strategy and further identify the factors influencing physician trust among internal migrants.

**Methods:**

This study used a sample of 1,200 respondents of Shanghai’s migrant population through an online survey (Wenjuanxing platform) from January 2021 to December 2021. K-means cluster analysis and multivariate logistic regression models were used to identify acculturation strategy patterns of internal migrants and the factors influencing their physician trust in China.

**Results:**

Among the 1,117 valid respondents, 85.5% were aged 18–39 years, 51.8% (579) were male, 74.8% (836) were married, and 62.9% (703) had completed university or junior college education, 62.1% (690) were covered by urban employee medical insurance, and 46.3% (517) had an annual income of 110,000–250,000 yuan. Through K-means clustering analysis, four clusters of acculturation strategy patterns were obtained: integration, assimilation, separation, and marginalization. When controlling for all significant socioeconomic and other covariates, compared to physician trust of the respondents adopting the separation acculturation strategy groups, the OR values of physician trust of the respondents adopting the integration and assimilation acculturation strategy groups were 1.979 (*p* < 0.01) and 1.585 (*p* < 0.01), respectively.

**Conclusion:**

Berry’s framework delineates four acculturation strategy patterns applicable to internal migrants in Chinese megacities, and the effects of these distinct patterns on trust in physicians have been demonstrated. This research provides valuable insights into the dynamics of doctor-patient relationships among internal migrants in China’s megacities. Therefore, it is advisable for the Chinese government to launch a combined effort from multiple stakeholders to adopt targeted interventions designed for specific demographic groups to foster greater trust in physicians among internal migrants.

## 1 Introduction

As the economy and society evolve and urbanization accelerates, internal migrants have emerged as one of the largest demographics in China. In Shanghai, for instance, the percentage of internal migrants has been steadily increasing, and their contributions to the city are also on the rise. However, this group has also introduced several challenges. One major issue is that the government struggles to create effective health service plans for internal migrants due to widespread barriers like information gaps and difficulties in emotional communication. This has led to a pressing demand for medical and health services among internal migrants, which is not being met by the limited medical resources available. Additionally, while these migrants contribute to urban life, they also seek to integrate socioeconomically and culturally into their new cities. Unfortunately, the existing urban–rural divide in China (1), along with restrictions related to registered residence (2) and medical insurance (3), fosters a sense of discrimination among this population. Especially, the hukou system in China, along with the urban–rural divide, plays a critical role in shaping the acculturation experiences and healthcare access of internal migrants. This system categorizes migrants as “outsiders,” which contributes to their marginalization and hinders their ability to assimilate into urban culture, as articulated in J.W. Berry’s model of acculturation (4). The presence of social stigma and limited access to essential services further complicates the acculturation process for these individuals. In terms of healthcare, the hukou system restricts access based on an individual’s registered domicile, thereby limiting these migrants’ ability to obtain affordable and high-quality healthcare in urban settings (5). Consequently, these migrants often depend on less comprehensive rural insurance, encounter higher out-of-pocket expenses, and exhibit a lower propensity to seek preventive care, which ultimately leads to adverse health outcomes. The urban–rural divide intensifies cultural and economic disparities, complicating the adaptation of migrants to urban lifestyles. Children of migrants face significant challenges within urban educational institutions (6), which can hinder their long-term integration. Although urban areas typically possess superior healthcare infrastructure, migrants frequently encounter obstacles in accessing care due to hukou restrictions and fragmented insurance systems. Furthermore, the stress associated with navigating these barriers adversely affects the mental health of internal migrants. In summary, the hukou system and the urban–rural divide present substantial obstacles to both acculturation and healthcare access, thereby perpetuating inequality and exclusion for internal migrants in China. Furthermore, the unmet demand for medical services contributes to a low perception and evaluation of healthcare, resulting in tensions between healthcare providers and patients. According to data from the Shanghai Health and Family Planning Commission in 2018, over 60% of medical disputes in 2017 involved the migrant population, which not only hindered their access to health services and overall health but also posed potential challenges for urban governance (7).

Numerous studies, both domestically and internationally, have examined methods to enhance the doctor-patient relationship among immigrant populations or transient populations, with a particular emphasis on the influence of demographic and socio-economic factors on physician trust and patient satisfaction. Physician trust refers to a patient’s positive expectation that their healthcare provider will prioritize their well-being and interests, particularly in vulnerable circumstances (8). This trust is a significant predictor of favorable health outcomes, encompassing patient satisfaction, proactive health-seeking behaviors, continuity of care, and adherence to treatment regimens (9). The establishment of trust is essential for fostering high-quality relationships between physicians and patients, whereas a lack of trust may result in suspicion and confrontational behavior from patients. The existing literature indicates that the assessment of physician trust can be approached through various methodologies: (1) Utilization of established trust scales, including the Wake Forest Physician Trust Scale (10), the Trust in Physician Scale (TPS) (11), Safran’s Personal Trust Scale (12), Kao’s Personal Trust Scale (13), and the Patient Trust in Medical Professionals Scale (14). (2) Observation of behavioral indicators, which encompass adherence to treatment regimens, the willingness to share personal health information, continuity of care (e.g., returning to the same physician) (15), and health-seeking behaviors (e.g., timely medical visits and follow-up appointments) (16). (3) Implementation of multidimensional composite scales that integrate factors such as communication quality, empathy, and shared decision-making (17). (4) Application of qualitative measurement techniques, including interviews and focus group discussions. However, there exists a relative paucity of research addressing the impact of acculturation. Prior investigations have indicated that the tension within doctor-patient relationships is correlated with the extent to which immigrants or internal migrants acculturate into mainstream society (18). Furthermore, factors such as language barriers, cultural dissimilarities, and social customs contribute to a diminished evaluation of medical and health services, thereby exacerbating the tension in doctor-patient interactions. In response to this issue, numerous scholars have explored the concept of acculturation, which is defined as the sustained and direct cultural interaction between two groups comprised of individuals from differing cultural backgrounds, resulting in variations in the existing cultural patterns of one or both parties (19). In this context, Berry (20) proposed a two-dimensional model of acculturation strategies, positing that adaptation to the host culture does not inherently necessitate the rejection of the culture of origin; rather, the attitudes of immigrants toward both the host culture and their culture of origin are independent of one another. Based on this framework, Berry identified four acculturation strategies: assimilation (the rejection of the culture of origin in favor of the host culture), separation (the maintenance of the culture of origin while discarding the host culture), marginalization (the simultaneous rejection of both cultures), and integration (the simultaneous acceptance of both cultures) (20). Prior literature suggests that the same acculturative strategy employed by immigrants may yield varying doctor-patient relationships, indicating a heterogeneity in these interactions. For instance, some researchers argue that immigrants who adopt separation strategies tend to exhibit greater distrust toward the healthcare system compared to those who embrace assimilation strategies, while individuals employing integration strategies are generally more trusting of healthcare providers and the healthcare system as a whole (21, 22).

In the realm of acculturation strategy classification, Lee et al. (23) utilized a two-dimensional scale established by J.W. Berry to examine the acculturation experiences of Korean Americans, identifying three primary strategies: integration, assimilation, and separation. Similarly, Renzaho et al. (24) categorized African immigrant children aged 3–12 in Australia into four distinct groups: traditional, integrated, assimilated, and marginalized. Furthermore, Ma and Xia (25) classified acculturation strategies among internal migrants in eight megacities in China into assimilation, separation, integration, and marginalization. Current scholarly consensus suggests that varying acculturation strategies significantly influence doctor-patient relationships. In this context, Tarn et al. (21) investigated the effects of ethnic matching, autonomy, acculturation, and religious beliefs on trust in physicians among Japanese and Japanese American immigrants. Their findings indicated that Japanese Americans who adopted assimilation strategies exhibited a higher level of trust in medical care compared to their Japanese counterparts. Additionally, two studies have demonstrated that collective ownership and autonomous preferences serve as mediating and moderating factors in the relationship between acculturation and medical distrust (21, 26). Ward and Kennedy (27) and Bhugra (28) both found that migrants using a marginalization strategy—rejecting both their native and host cultures—experience the poorest psychological adjustment and lowest sociocultural competence. They face high stress, anxiety, and alienation, often leading to social isolation. This disconnection fosters distrust toward their own community, the host society, and healthcare systems, exacerbating their challenges in adapting to the new environment (27, 28). Nesdale and Mak (29) determined that individuals who utilize a marginalization strategy are subjected to the most pronounced adverse consequences, such as diminished self-esteem and inadequate psychological adjustment, thereby exacerbating their feelings of distrust.

As opposed to cross-border migration, some scholars also investigated the internal migration using Berry’s acculturation strategies. Gui et al. (30) conducted a questionnaire survey involving 787 migrant workers and discovered that J.W. Berry’s acculturation strategy model, which is predicated on the identity of international immigrants, is applicable to seasonal migrant workers. The findings indicate that the integration strategy is most beneficial for well-being in terms of both social identity and place identity, whereas marginalization is the least advantageous. These results align with prior research on the acculturation of international immigrants. Using Berry’s framework, Yuan et al. (31) studied 680 migrant children in two types of Chinese schools over 1 year. Results showed improved psychological adaptation in both, but only public school students improved in sociocultural adaptation. Identification with the culture of origin decreased in public schools but increased in migrant schools, while host culture identification showed opposite trends (32). Nagla (32) found that India’s population migration is primarily rural–urban, driven by economic opportunities (31). In accordance with J.W. Berry’s acculturation framework, the patterns of urban–rural and interstate migration in India suggest that interstate migrants may employ integration strategies. The migration of women is predominantly associated with familial obligations, leading them to favor separation strategies that allow for the preservation of their original cultural identities. In contrast, male migrants, motivated predominantly by economic factors, tend to adopt strategies of assimilation or integration. This observation challenges Berry’s assertion, which emphasizes adaptation primarily within psychological and socio-cultural contexts. Nevertheless, the majority of research concerning Berry’s acculturation strategy model has predominantly concentrated on international immigration. In contrast, there is a notable deficiency in the literature that utilizes this theoretical framework to examine physician trust, particularly among domestic migrants within the Chinese context. Consequently, this study employed the four-type acculturation strategy theory as a foundational framework to ascertain its applicability to internal migrants in China and to identify the determinants that influence physician trust. Enhancing doctor-patient relationships and optimizing governmental population management and public services in China, as well as in other developing nations with substantial indigenous populations, holds significant practical importance.

Based on Berry’s theory of acculturation strategies, we proposes the following hypotheses to confirm:

*Hypothesis 1*: Internal migrants in China exhibit various patterns of acculturation strategies, which supports the theory of Berry’s acculturation theory.

*Hypothesis 2*:

2a: The group that employs an integrated acculturation strategy demonstrates a greater degree of trust compared to the group that utilizes a separation strategy.

2b: The group that employs an assimilated acculturation strategy demonstrates a greater degree of trust compared to the group that utilizes a separation strategy.

2c: The group that employs a marginalized acculturation strategy demonstrates a lower degree of trust compared to the group that utilizes a separation strategy.

## 2 Data and methods

### 2.1 Data sources

The research team collected 1,200 samples of Shanghai’s migrant population through an online survey (Wenjuanxing platform) from January 2021 to December 2021. A systematic sampling technique was employed to choose qualified participants. Individuals were classified as internal migrants and included in the research if they met the following criteria: (1) they were 18 years of age or older, and (2) they were not originally from Shanghai but had received legal permanent or temporary residency permits from the Migrant Population Management Office for a minimum of 6 months. The online survey was developed in collaboration with Shanghai community health centers and migrant associations to ensure cultural relevance and accessibility. These stakeholders contributed to question phrasing, advised on distribution through migrant-frequented WeChat groups, designed participation incentives, and helped disseminate the questionnaire via community health workers and migrant-led organizations. They also translated materials into regional dialects to minimize language barriers, ensuring effective outreach to hard-to-access populations.

The online questionnaire is set to be answered through a single IP address. The questionnaire will be excluded once is detected: (1) Completed within less than 200 s (15, 1.25%); (2) Providing repeated answers or some form of answer (45, 3.75%); (3) having 5% or more missing values (10, 0.83%); (4) having editing errors (19, 1.57%), in total, 1,111 eligible participants were included in the final analyses (response rate is 92.6%).

This study was carried out in accordance with the Shanghai University of Medicine and Health Sciences Institutional Review Board for the Protection of Human Subjects on March 5, 2019 (No. 2019-gskyb-02-372424198012222511). All the participants provided written informed consent.

### 2.2 Methods

#### 2.2.1 Measurements

The measurement of physician trust adopts a physician trust scale based on medical quality management -developed by the research team (33). This scale contains 12 items, divided into four dimensions: environmental quality trust, interpersonal relationship quality trust, technical quality trust, and management quality trust. The assignment of each item is in the form of a Likert scale, ranging from “completely disagree” (assigned a score of 1) to “completely agree” (assigned a score of 5). The higher the score, the higher the level of physician trust. For the convenience of research, the average trust score is divided into two categories: “good (=1)” and “poor” (=0). In this study, the internal consistency coefficients of the four dimension scales were 0.764, 0.831, 0.734, and 0.832, respectively. The internal consistency coefficient of the total physician trust scale was 0.876.

The measurement of acculturation strategy adopts a scale developed by Miao and Xiao (61), based on the Berry (34) Acculturation Scale and the Vancouver Index of Acculturation (VIA) Scale (35). it consists of two dimensions and eight items in total, with 4 items measuring attitudes toward the separation of local culture, and 4 items measuring attitudes toward the maintenance of culture of origin. The assignment of each item ranges from “strongly disagree” (assigned as 1 score) to “strongly agree” (assigned as 5 score), with higher scores indicating lower adaption of the local culture and greater maintenance of the original culture, respectively. In this study, the internal consistency coefficients of the two dimensions were 0.809 and 0.797, respectively, and the internal consistency coefficient of the total scale of acculturation strategy was 0.797.

The Years of Residency (YOR) was divided into four periods: “6 months-1 years,” “1–5 years,” “5–10 years” and “> = 10 years”.

Previous research has examined various metrics of health service usage, such as the frequency of hospital admissions, doctor appointments, emergency room visits, and physical examinations (36, 37), we measured health service utilization using two questions, in the study, namely “How frequently did you undergo physical examinations in the past year?” “How frequently did you see a doctor in the past year?” Additionally, as health service utilization is also determined by healthcare-seeking behaviors (38, 39), we added another question to indirectly assess how health services are used: “In the past year, what has been your first-choice medical institution when you need to see a physician?” The first two questions are rated from 0 (no) to 3 (3 times or more) points, with a higher score indicating greater utilization of health services. For the question on medical institution preference, institutions were mainly classified into “hospitals” and “primary health service institutions.”

The number of type I and type II diseases that a respondent had was measured using a single question. Type I diseases include a total of 10 chronic conditions, which are asthma, back pain, hypertension, high cholesterol, diabetes, allergies, migraines, ulcers, bronchitis, and arthritis. The responses were rated from 1 to 3 (1 point, no disease; 2 points, one disease; and 3 points, two or more diseases). Type II diseases comprised three critical diseases (heart disease, cancer, and blood disease, other serious illnesses such as thyroid disorders, stroke, uremia, septicemia, amyotrophic lateral sclerosis, and Alzheimer’s disease are not included in the analysis due to their low occurrence in the study population aged 18 to 60 years). Responses were rated from 1 to 2 points (1 point, no disease; 2 points, one or more disease).

Health status was assessed based on the self-rated health (SRH) status scale developed by the research team (14), which consists of five items that cover physical health, mental health, and social support. Each item is rated from 1 (very good) to 5 (very poor). A lower mean score for the five items indicates a more favorable health status. For convenience, the mean score was dichotomized into two categories (: good and poor). The internal consistency coefficients of the total scale of self-reported health before and after entry in this study were 0.819 and 0.910, respectively.

The classifications and measurements of the main variables of interest are presented in Table 1.

**Table 1:** The classification and measures of scoring variables.

Variables	How to measure	Type
Physician trust	1 = Good; 0 = Bad	Categorical
Years of residency	1 = 6 months-1 year; 2 = 1–5 years; 3 = 5–10 years; 4 = ≥10 years	Categorical
Age	1 = ≤39 old years; 2 = ≥40–60 old years	Categorical
Gender	1 = Male; 0 = Female	Categorical
Marital status	1 = Yes; 0 = No	Categorical
Educational level	1 = High school or below; 2 = University (including junior college); 3 = Graduate or above	Categorical
Professional type	1 = Unemployed; 2 = Civil sevant; 3 = Company or enterprise; 4 = Migtant workers; 5 = Skilled worker	Categorical
Place of birth^a^	1 = Shanghai; 2 = Eastern China (excluding Shanghai); 3 = Central china; 4 = Western China; 4 = Northeastern China	Categorical
Insured type	1 = UEBMI; 2 = URRBMI; 3 = FI; 4 = CMI or others	Categorical
Annual income	1 = CNY100,000 or below; 2 = CNY110,000 -CNY250,000; 3 = CNY260,000 -CNY400,000; 4 = CNY410,000 -CNY600,000; 5 = CNY600,000 or more	Categorical
The frequency of physical examinations in the past year	1 = None; 2 = once; 3 = Two times; 4 = Three times or more	Categorical
The frequency of physician visits physician in the past year	1 = None; 2 = once; 3 = Two times; 4 = Three times or more	Categorical
The preferred health-care institution	1 = Hospitals; 2 = Primary care institutions	Categorical
The number of disease І you have	0 = None of the above; 2 = One; 2 = Two or more	Categorical
The number of disease II you have	0 = None of the above; 1 = One or more	Categorical
SRH	1 = Favorable; 0 = Not favorable	Categorical

Eastern region includes 10 provinces (municipalities): Beijing, Tianjin, Hebei, Liaoning, Shanghai, Jiangsu, Zhejiang, Fujian, Shandong, Guangdong and Hainan; Central region includes 6 provinces: Shanxi, Anhui, Jiangxi, Henan, Hubei and Hunan; Western region includes 12 provinces (autonomous regions, municipalities): Inner Mongolia, Guangxi, Chongqing, Sichuan, Guizhou, Yunnan, Tibet, Shaanxi, Gansu, Qinghai, Ningxia and Xinjiang; North east China (also known as Inner Manchuria) includes Heilongjiang, Jilin and Liaoning. UEBMI, urban employee basic medical insurance; URRBMI, urban and rural residents basic medical insurance; FI, free insurance; CMI, Commercial medical insurance; SRH, self-rated health.

#### 2.2.2 Analysis methods

Initially, a Kolmogorov–Smirnov (K–S) test was employed to assess the normality of the data distribution. Following this, a descriptive analysis was performed, which encompassed an examination of demographic and socio-economic characteristics, trust in physicians, healthcare service utilization, types of diseases, and other pertinent variables.

Subsequently, a one-way analysis of variance (ANOVA) was conducted to analyze the dependent variable of physician trust in relation to independent variables encompassing individual characteristics, including demographic and socio-economic factors, healthcare service utilization, disease types, and self-rated health (SRH). An independent samples *t*-test was utilized for binary independent variables, while one-way ANOVA was applied for Mult categorical independent variables. Statistically significant covariates identified through one-way ANOVA or *t*-tests were retained for inclusion in subsequent multivariate logistic regression analyses.

An exploratory K-means cluster analysis was undertaken to determine whether distinct patterns of acculturation strategies, as posited by Berry’s acculturation strategy theory, could be discerned from the collected data. Prior to this analysis, all variables were normalized to reduce the influence of variations in standard deviations or means. Continuous variables were transformed into z-scores (mean = 0; standard deviation = 1) to accommodate the diverse scales of measurement. The selection of variables was informed by the acculturation strategy framework, which highlights the adaptation to local culture and the maintenance of the original culture as fundamental components of the proposed assimilation patterns.

Recognizing that covariates such as age (40), gender (41), marital status (42), education level (42), income, healthcare service utilization (43), and the number of chronic diseases (40, 44) may influence physician trust, this study incorporated these factors as covariates and utilized them as controlled variables in the multivariate logistic regression model. This approach facilitated the establishment of an unadjusted null model (Model 1) and several adjusted models (Models 2, 3, and 4). Model 2 represented Model 1 adjusted for demographic and socio-economic characteristics, Model 3 was Model 2 further adjusted for healthcare service utilization, and Model 4 included the variables of SRH status before and after relocation to Shanghai, as well as the number of type I and type II diseases.

To evaluate multicollinearity among the independent variables, the Variance Inflation Factor (VIF) index was employed. A VIF value of less than 5 indicated that the variable was acceptable for inclusion in the regression analysis. Additionally, to assess the model’s goodness of fit and predictive capability, Receiver Operating Characteristic (ROC) curves and Area Under the Curve (AUC) indices were utilized to evaluate the model’s fit.

Community feedback sessions were held to contextualize quantitative findings, particularly on trust dynamics, ensuring alignment with on-the-ground realities. These sessions involved internal migrant focus groups or healthcare providers and focused on briefly describe topics, e.g., interpreting physician trust scale results.

### 2.3 Statistical software

In this study, the statistical analyses were conducted using Stata version 15.1. The determination of statistical significance for the t-test, one-way ANOVA, and logistic regression was established at a threshold of *p* < 0.05 (two-tailed).

## 3 Results

### 3.1 Respondent’s characteristics analyses and various patterns of acculturation strategies

Among the 1,117 respondents who submitted valid responses, 85.5% were aged 18–39 years, 51.8% (579) were male, 74.8% (836) were married, and 62.9% (703) had completed university or junior college education, 62.1% (690) were covered by urban employee medical insurance, and 46.3% (517) had an annual income of 110,000–250,000 yuan (Table 1).

After conducting K-means clustering analysis, four main patterns were identified: integration, assimilation, separation, and marginalization, with a silhouette score of 0.377, suggesting a moderate quality of clustering (see a Supplementary file). The distribution of the clustering variables for each group is presented in Table 2.

**Table 2:** Mean and variance statistics of different acculturation strategy models (*n* = 1,117).

Acculturation	Integration (*n* = 302)	Assimilation (*n* = 212)	Separation (*n* = 177)	Marginalization (*n* = 426)
Adaptation to the culture of the destination^a^	16.25(3.51)	13.75(4.01)	29.00(4.73)	24.56(3.01)
Maintain the culture of one’s hometown	29.05(3.97)	17.41(4.19)	31.81(3.63)	22.96(3.42)

^aReverse encoding.^

### 3.2 Bivariate analysis

The results of the t/ANOVA test indicate that several variables exhibit a significant correlation with physician trust. These variables include: acculturation strategy type (*χ*^2^ = 27.47, *p* < 0.001), years of residence (YOR) (*χ*^2^ = 40.39, *p* = 0.002), marital status (*t* = −3.115, *p* = 0.001), profession type (*χ*^2^ = 16.35, *p* = 0.003), annual income (*χ*^2^ = 14.303, *p* = 0.002), frequency of physician visits in the past year (*χ*^2^ = 9.66, *p* = 0.044), self-rated health (SRH) prior to floating (*t* = −3.64, *p* < 0.001), and SRH following floating (*t* = −1.149, *p* < 0.001). Conversely, the following variables did not demonstrate a significant correlation with physician trust: age (*t* = 1.6566, *p* = 0.0979), gender (*t* = −0.1837, *p* = 0.870), education level (*χ*^2^ = 1.1395, *p* = 0.768), insurance type (*χ*^2^ = 8.0746, *p* = 0.089), place of birth (*χ*^2^ = 4.6213, *p* = 0.363), preferred healthcare institution (*t* = −0.1923, *p* = 0.847), frequency of physical examinations in the past year (*χ*^2^ = 6.62, *p* = 0.084), the number of type-I diseases (*χ*^2^ = 3.72, *p* = 0.156), and the number of type-I diseases (*t* = 1.3242, *p* = 0.1857). For further details, please refer to Table 3.

**Table 3:** Results of the descriptive and univariate analysis (*n* = 1,117).

Variables	*n* (%)	Physician trust		*χ*^2^/*t*/*p*-values
		Good	Poor	
		N1 (%)	N2 (%)	
Acculturation strategy patterns (*n* = 1,111)				χ^2^ = 27.472 *p* < 0.001
Integration	302(27.04%)	263(87.4%)	38(12.6%)	
Assimilation	212(18.98%)	175(82.55%)	37(17.45%)	
Separation	177(15.85%)	128(72.32%)	49(27.68%)	
Marginalization	426(38.14%)	310(72.77%)	116(27.23%)	
YOR (*n* = 1,110)				*χ*^2^ = 40.3915 *p* = 0.022
<1 year	187(16.7%)	140(74.9%)	47(25.1%)	
1–5 years	465(41.6%)	378(81.3%)	87(18.7%)	
5–10 years	241(21.6%)	184(76.3%)	57(23.7%)	
≥10 years	217(19.4%)	168(77.4%)	49(22.6%)	
Age (*n* = 1,113)				*t* = 1.6566.*p* = 0.0979
≤39 old years	955(85.5%)	757(79.3%)	198(20.7%)	
40–60 old years	158(14.1%)	116(73.4%)	42(26.6%)	
Gender (*n* = 1,114)				*t* = −0.1837 *p* = 0.870
Male	579(51.8%)	453(78.2%)	126(21.8%)	
Female	535(47.9%)	421(78.7%)	114(21.3%)	
Marital status (*n* = 1,116)				*t* = −3.1153 *p* = 0.001
No	280(25.1%)	200(71.4%)	80(28.6%)	
Yes	836(74.8%)	676(80.9%)	160(19.1%)	
Educational level (*n* = 1,117)				*χ*^2^ = 1.1395 *p* = 0.768
High school or below	96(8.6%)	76(79.2%)	20(20.8%)	
University (including junior college)	157(14.1%)	121(77.1%)	35(22.9%)	
Graduate or above	703(62.9%)	557(79.2%)	146(20.8%)	
	161(14.4%)	122(75.8%)	39(24.2%)	
Profession type (*n* = 1,116)				*χ*^2^ = 16.3530 *p* = 0.003
Unemployment (or retired)	97(8.7%)	68(70.1%)	29(29.9%)	
Civil servant	86(7.7%)	56(65.1%)	30(34.9%)	
Company (or enterprise)	735(65.9%)	595(81.0%)	140(19.1%)	
Migrant workers	82(7.4%)	67(81.7%)	15(18.3%)	
Skilled workers	116(10.4%)	90(77.6%)	26(22.4%)	
Place of birth (*n* = 1,114)				*χ*^2^ = 4.6213 *p* = 0.363
Shanghai	112(10%)	94(83.9%)	18(16.1%)	
Eastern China (excluding Shanghai)	558(50%)	442(79.2%)	116(20.8%)	
Central China	264(23.6%)	203(76.9%)	61(23.1%)	
Western China	123(11%)	90(73.2%)	33(26.8%)	
North East China	57(5.1%)	44(77.2%)	13(22.8%)	
Annual income (*N* = 1,111)				*χ*^2^ = 14.3033 *p* = 0.002
CNY100,000 or below	288(25.8%)	204(70.8%)	84(29.2%)	
CNY110,000 –CNY250,000	517(46.3%)	423(81.8%)	94(18.2%)	
CNY260,000 –CNY400,000	222(19.9%)	178(80.2%)	44(19.8%)	
CNY410,000 –CNY600,000	55(4.9%)	44(80%)	11(20%)	
CNY600,000 or more	30(2.7%)	25(83.3%)	5(16.7%)	
Insured type (*n* = 1,112)				*χ*^2^ = 8.0746 *p* = 0.089
UEBMI	690(62.1%)	542(78.6%)	148(21.4%)	
URRMI	179(16.1%)	148(82.7%)	31(17.3%)	
FI	41(3.7%)	31(75.6%)	10(24.4%)	
CMI or others	141(12.6%)	111(78.7%)	30(21.3%)	
No insured	61(5.5%)	40(65.6%)	21(34.4%)	
The frequency of physician visits physician in the past year (*n* = 1,093)				*χ*^2^ = 9.6600 *p* = 0.044
None	237(21.2%)	173(73%)	64(27%)	
Once	367(32.9%)	290(79%)	77(21%)	
Two time	305(27.3%)	255(83.6%)	50(16.4%)	
Three times or more	184(16.5%)	140(76.1%)	44(23.9%)	
The frequency of physical examinations in the past year (*n* = 1,101)				*χ*^2^ = 6.6410 *p* = 0.084
None	247(22.1%)	185(74.9%)	62(25.1%)	
Once	616(55.1%)	477(77.4%)	139(22.6%)	
Two time	183(16.4%)	153(83.6%)	30(16.4%)	
Three times or more	55(4.9%)	47(85.5%)	8(14.5%)	
The preferred health-care institution (*n* = 924)				*t* = −0.1923 *p* = 0.847
Hospitals	698(62.5%)	558(79.9%)	140(20.1%)	
Primary care institutions	226(20.2%)	182(80.5%)	44(19.5%)	
The number of type-І disease^a^ (*n* = 1,113)				*χ*^2^ = 3.7138 *p* = 0.156
None of above	698(62.7%)	537(76.9%)	161(23.1%)	
One	342(30.7%)	280(81.9%)	62(18.1%)	
Two or more	73(6.6%)	55(75.3%)	18(24.7%)	
The number of type-II disease^b^ (*n* = 1,108)				*t* = 1.3242 *p* = 0.1857
None of above	1,036(93.5%)	817(78.9%)	219(21.1%)	
One or more	72(6.5%)	52(72.2)	20(27.8%)	
SRH before floating (*n* = 1,115)				*t* = −3.6407 *p* < 0.001
Favorable	1,060(95.1%)	844(79.6%)	216(20.4%)	
Not favorable	55(4.9%)	30(54.6%)	25(45.5%)	
SRH after floating (*n* = 1,117)				*t* = −11.1487 *p* < 0.001
Favorable	902(80.8%)	777(86.1%)	125(13.9%)	
Not favorable	215(19.2%)	99(46.1%)	116(53.9%)	

^a^Type-І disease includes 10 common chronic health conditions, namely asthma, back pain, hypertension, hyperlipidemia, diabetes, allergies, migraine, ulcers, bronchial inflammation, and arthrit; ^b^type-II disease encompassed three common critical diseases: heart disease, blood-related cancers (e.g., leukemia), and other cancers. SRH, self-rated health; UEBMI, urban employee basic medical insurance; URRBMI, urban and rural residents basic medical insurance; FI, free insurance; CMI, commercial medical insurance.

### 3.3 Multivariate logistic regression analysis

Multicollinearity testing revealed that the Variance Inflation Factors (VIFs) for the independent variables incorporated in the logistic regression model ranged from 1.04 to 1.44, suggesting that multicollinearity is not present.

The findings from unadjusted Model 1 indicate that the odds ratio (OR) for the integration acculturation type was 2.582 (*p* < 0.001), while the OR for the assimilation acculturation type was 1.811 (*p* < 0.001). These results suggest that individuals who adopted the integration acculturation type demonstrated were 2.582 and 1.811 times more likely to report a high level of physician trust compared to those who identified with the separation acculturation type.

When the covariates of YOR, age, gender, marital status, profession type, insurance type, Educational level, annual income, the frequency of doctor visits in the past year, the frequency of physical examinations in the past year, and SRH before and after floating were controlled for, except of assimilation strategy pattern (all increased from model 1 to model 4), the association between acculturation strategy patterns and physician trust initially increased (i.e., Models 2 and 3), but subsequently decreased (i.e., Model 4). In Model 4, the OR values of the respondents adopting the integration and assimilation acculturation strategy groups were 1.979 (*p* < 0.01) and 1.585 (*p* < 0.01), respectively. That is, respondents in the first-generation assimilation groups were 1.585–1.979 times more likely to report a high level of physician trust than those who adopt the separation acculturation strategy group.

Regarding the covariates that could influence physician trust, the respondents who aged 40–60 old years were 41.3% less likely to report a high degree of trust in physicians compared to those under 40 old years (OR = 0.587, *p* < 0.01), and those who had university and graduate degrees or above were 53.3 and 57.3%, respectively, less likely to express a high degree of trust in physicians compared to those who completed junior high school education or below (OR = 0.467, *p* < 0.05; OR = 0.427, *p* < 0.05). By contrast, the respondents who participated in URRMI were 2.214 times more likely to indicate a high level of trust in physicians compared compared to those who had not been insured at all (OR = 2.214, *p* < 0.05). Moreover, the respondents who had annual incomes of 110,000–250,000 yuan and 260,000–400,000 yuan were 1.916 and 1.798 times, respectively, more likely to indicate a high level of trust in physicians compared to individuals with an annual income of 100,000 yuan or less (OR = 1.916, *p* < 0.001; OR = 1.798, *p* < 0.05). Relative to respondents who did not see a physician, those who consulted a physician two or more times were 2.058 times more likely to express a high level of trust in physicians (OR = 2.058, *p* < 0.001). In a similar vein, individuals who indicated they had good health both prior to and after moving to the city were 2.283 and 6.363 times more likely, respectively, to express a high level of trust in their physicians compared to those who reported poor health (OR = 2.283, *p* < 0.001; OR = 6.363, *p* < 0.001). In Model 4 (Table 4), the covariates YOR, gender, marital status, profession type, and the frequency of physical examinations in the past year were not significant.

**Table 4:** Results of multivariate logistic regression analysis.

Independent variable	Model 1^a^	Model 2^b^	Model 3^c^	Model 4^d^
Acculturation strategy type				
Separation	1.000	1.000	1.000	1.000
Integration	2.582***	2.665***	2.686***	1.979**
	(1.739–3.833)	(1.765–4.022)	(1.761–4.096)	(1.253–3.126)
Assimilation	1.811**	1.730**	1.635**	1.585**
	(1.206–2.818)	(1.263–2.751)	(1.251–2.291)	(1.520–1.908)
Marginalization	1.023	1.140	1.208	1.076
	(0.736–1.422)	(0.807–1.608)	(0.846–1.725)	(0.733–1.581)
YOR				
<1 year			1.000	1.000
1–5 years		1.160	1.204	1.501
		(0.811–1.658)	(0.832–1.741)	(0.005–2.244)
5–10 years		0.757	0.756	0.766
		(0.501–1.144)	(0.495–1.153)	(0.489–1.200)
≥10 years		0.952	1.024	1.390
		(0.608–1.490)	(0.646–1.623)	(0.847–2.283)
Age				
≤39 years old		1.000	1.000	1.000
40–60 years old		0.621**	0.607**	0.587**
		(0.419–0.919)	(0.407–0.907)	(0.448–0.953)
Gender				
Male			1.000	1.000
Female		0.945	0.965	1.051
		(0.728–1.227)	(0.736–1.265)	(0.783–1.410)
Marital status				
No			1.000	1.000
Yes		1.680***	1.556**	1.005
		(1.228–2.299)	(1.129–2.143)	(0.700–1.444)
Occupational type				
Unemployment (or retirement)			1.000	1.000
Civil servant		0.667	0.700	0.654
		(0.365–1.218)	(0.375–1.307)	(0.329–1.299)
Company (or enterprise)		1.160	1.163	0.874
		(0.723–1.860)	(0.712–1.900)	(0.523–1.461)
Migrant workers		1.517	1.584	1.410
		(0.812–2.832)	(0.841–2.983)	(0.704–2.823)
Skilled workers		0.977	1.114	0.845
		(0.553–1.726)	(0.607–2.045)	(0.446–1.602)
Insurance type				
No insuranced			1.000	1.000
UEBMI		1.421	1.700	1.959
		(0.749–2.696)	(0.861–3.357)	(0.896–4.284)
URRMI		1.893	2.048*	2.214*
		(0.996–3.599)	(1.031–4.069)	(1.004–4.878)
FI		1.529	1.609	1.713
		(0.634–3.691)	(0.635–4.075)	(0.639–4.592)
CMI or others		1.573	1.791	2.345
		(0.805–3.073)	(0.878–3.653)	(0.939–4.292)
Educational level				
Junior or below			1.000	1.000
High school		0.792	1.142	0.885
		(0.431–1.457)	(0.598–2.183)	(0.448–1.745)
University (including junior colleges)		0.498*	0.717*	0.467*
		(0.273–0.910)	(0.377–0.965)	(0.233–0.937)
Graduate or above		0.454*	0.684	0.427*
		(0.225–0.918)	(0.322–1.453)	(0.186–0.982)
Annual income				
CNY100,000 or below			1.000	1.000
CNY 110,000–CNY 250,000		1.942***	1.914***	1.916***
		(1.345–2.804)	(1.311–2.795)	(1.277–2.875)
CNY 260,000–CNY 40,000		1.780**	1.615*	1.798*
		(1.142–2.776)	(1.015–2.571)	(1.088–2.974)
CNY 410,000–CNY 600,000		1.819	1.486	1.599
		(0.939–3.525)	(0.736–3.000)	(0.752–3.401)
CNY 600,000 or more		2.119	2.019	2.192
		(0.823–5.457)	(0.749–5.440)	(0.796–6.036)
The frequency of doctor visits in the past year				
None			1.000	1.000
Once			1.315	1.509
			(0.910–1.898)	(0.805–2.267)
Two times			1.594*	2.058***
			(1.061–2.395)	(1.335–3.173)
Three times or more			1.041	1.222
			(0.676–1.602)	(0.763–1.959)
The frequency of physical examinations in the past year				
None			1.000	1.000
Once			0.770	0.765
			(0.533–1.113)	(0.521–1.123)
Two times			1.152	1.042
			(0.710–1.869)	(0.611–1.780)
Three times or more			1.200	0.945
			(0.557–2.585)	(0.429–2.083)
SRH before floating				
Favorable				1.000
Not favorable				2.283***
				(1.405–3.711)
SRH after floating				
Favorable				1.000
Not favorable				6.363***
				(4.592–8.815)
Constant term	2.612***	1.358	0.749	0.164***
	(1.981–3.444)	(0.676–2.728)	(0.339–1.656)	(0.0630–0.428)
Pseudo *R*^2^	0 0.0245	0.0613	0 0.0686	0.1641
Observation quantity	1,117	1,093	1,060	1,058

^aUnadjusted crude model; bMultivariate model adjusted for demographic and socioeconomic characteristics, as well as years of residency; cMultivariate model adjusted for the frequency of physical examinations in the past year, the frequency of physical examinations in the past year based on model 2; dMultivariate model adjusted for SRH before floating and SRH after floating based on Model 3; SRH, self-rated health; UEBMI, urban employee basic medical insurance; URRBMI, urban and rural residents basic medical insurance; FI, free insurance; CMI, Commercial medical insurance.^

*p < 0.05, **p < 0.01, ***p < 0.001.

**Table 5:** Comparison of model validation indexes of four models.

Model	Model 1	Model 2	Model 3	Model 4
AIC value	1144.41	1115.71	1090.07	987.76
BIC value	1164.48	1240.62	1244.01	1151.58
AUC value	0.593	0.667	0.672	0.760

### 3.4 Model validation

**Figure 1:**
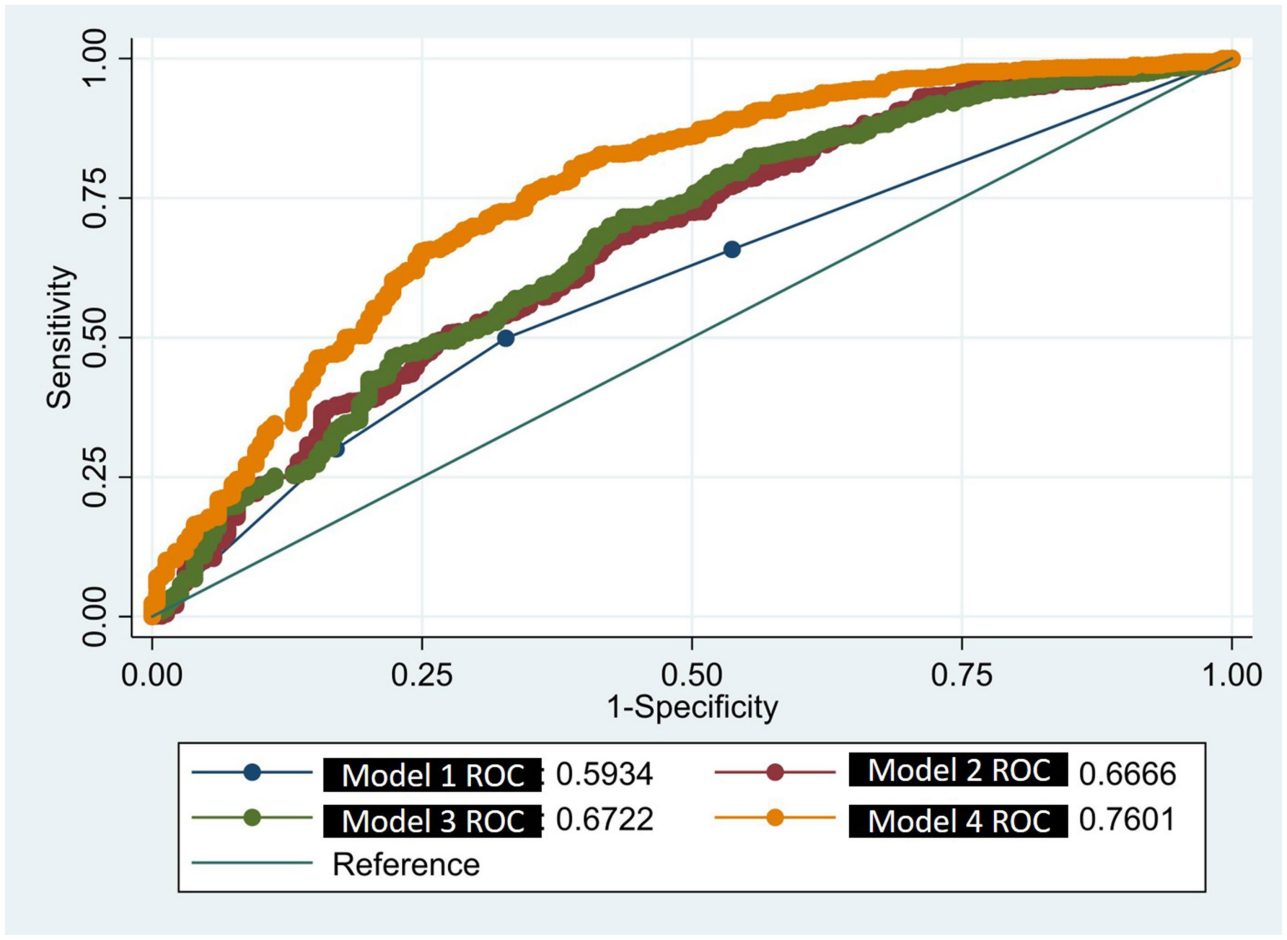
Comparison of ROC area of four different models.

In comparison to Model 3, which excludes health status and has an area under the curve (AUC) of 0.672, and Model 2, which omits health service utilization with an AUC of 0.667, as well as Model 1, which does not account for demographic, socio-economic, health service utilization, and health status covariates and has an AUC of 0.593, Model 4 demonstrates the highest AUC value of 0.760. Regarding model fit, the Akaike Information Criterion (AIC) values for the four models are 1144.41, 1115.71, 1090.07, and 987.76, respectively (refer to Table 5 and Figure 1). Consequently, Model 4 was chosen as the final model.

## 4 Discussion

This research employs k-means clustering and multivariate logistic regression analyses to substantiate the applicability of Berry’s acculturation theory to the doctor-patient relationship among internal migrants within the Chinese context. The H1 is validated. Moreover, when controlling for all covariates, it was found that respondents who adopted integration or assimilation strategies exhibited significantly higher levels of trust in physicians compared to those who identified with the separation strategy, thereby confirming H2a and H2b. In contrast, that respondents who adopted marginalization acculturation strategies exhibit no salient physician trust compared to those who identified with the separation strategy, thus rejecting H2c. Furthermore, various factors that significantly impacted trust in physicians among internal migrants in China were identified, including age, level of education, annual income, the frequency of medical visits over the past year, and self-rated health status both prior to and following population mobility.

Firstly, H1 was confirmed revealing that internal migrants in Shanghai can be categorized into four distinct groups based on their acculturation strategies: integration, assimilation, separation, and marginalization. This suggests an extension of the Berry’s acculturation theory to encompass both international immigrants and internal migrants. Migrants, whether they are relocating from abroad or moving within a country, encounter various challenges related to assimilation, acculturation, and the establishment of trust with healthcare providers as they navigate a new environment and engage with the healthcare system. The segmented assimilation theory further demonstrates that varying patterns of acculturation strategies, along with other determinants such as educational attainment and income levels, can result in distinct patterns of segmented assimilation.

Secondly, the internal migrant demographic that employs integration or assimilation as an acculturation strategy exhibits a higher level of trust in physicians compared to those who adopt a separation strategy. These results align with the findings of Tarn et al. (21). This phenomenon can be attributed to the floating population that embraces these two acculturation strategies, which has demonstrated an adaptation to the local culture. Such adaptation is associated with diminished behavioral and emotional responses, often referred to as acculturation pressure, stemming from the challenges of acclimating to a new cultural context (45). Consequently, this leads to an elevated level of trust in the local healthcare system and healthcare professionals, particularly in comparison to the floating population that employs a separation strategy. Furthermore, research indicates that internal migrants who are strongly acculturated tend to engage in more effective communication and interaction with healthcare providers in their area of residence, including overcoming language barriers, which fosters a sense of trust (46, 47). Whittal et al. (48) also suggested that immigrants who adopt integrative and assimilative acculturation strategies may influence their perceptions of doctor-patient relationships, thereby enhancing trust between healthcare providers and patients (48). These findings highlight the need for targeted interventions to improve physician trust among internal migrants adopting separation or marginalization strategies. Community stakeholders, including migrant representatives and local health bureaus, co-developed interventions such as cultural competency training for physicians and multilingual health literacy campaigns, directly informed by our results. Such initiatives could mitigate acculturation-related barriers by fostering mutual understanding between healthcare providers and migrant populations.

Thirdly, various demographic and socioeconomic factors, including age (particularly individuals aged 40 to 60), educational attainment (specifically those holding undergraduate or graduate degrees), annual income ranging from CNY 110,000 to CNY 400,000, and health service utilization indicators such as the frequency of medical visits (two or more in the preceding year), as well as health outcomes characterized by favorable self-rated health (SRH) prior to or following migration, have been identified as significant determinants of trust in physicians. These findings are consistent with the results of previous studies (9, 21, 43, 44, 49–57). It is noteworthy that the causal relationship between SRH and trust in physicians remains ambiguous, as some research suggests that trust in physicians may influence SRH. This ambiguity arises from the observation that a lower level of interpersonal trust between healthcare providers and patients can lead to reduced utilization of healthcare services, which may result in delayed treatment and subsequently lower SRH (58).

This study presents several limitations that warrant consideration. Firstly, the sample population consisted predominantly of young migrants, with an average age of under 40 years (85.80%). The generalizability of specific conclusions derived from this study may be affected by the heterogeneity present between younger and older internal migrant groups concerning various socioeconomic factors, such as educational attainment, income, health service utilization, acculturation, and self-assessed health status. The study has demonstrated that these factors significantly influence trust in physicians, which may consequently restrict the applicability of the findings and the scope of the policy recommendations proposed. Moreover, this demographic characteristic also suggests that intergenerational and family structure factors, which significantly influence the acculturation processes of internal migrants, may not have been adequately represented. For instance, the acculturation experiences of second-generation internal migrants often differ from those of first-generation migrants. Furthermore, all else being equal, families with two parents typically exhibit higher income levels compared to single-parent families. Consequently, future research should incorporate intergenerational and family structure variables to assess their impact on acculturation strategies and physician trust. Additionally, it is imperative to broaden the scope of the survey with longitudinal data to investigate the evolving trends associated with Berry’s acculturation strategy theory among internal migrants, thereby enhancing the generalizability of the study’s findings. Secondly, the dependent variable, physician trust, was measured through self-reported data, which may introduce social desirability and recall bias. Future studies should formulate questions in an unbiased and neutral manner, or utilize prospective studies to establish specific time frames or reference points. This approach can alleviate the pressure to provide confirmation and assist participants in recalling events with greater accuracy. Thirdly, the decision to dichotomize self-rated health (SRH) and physician trust into two categories was made primarily for clinical relevance and ease of interpretation, despite the potential loss of some granular information. Employing multiple categories or performing sensitivity analyses is typically advantageous for capturing more nuanced variations and ensuring the robustness of findings in future research endeavors. Finally, based on current research on migration (59, 60), it is recommended to incorporate psychological and cultural elements like psychological adaptation, cultural distance, and perceived discrimination, as these may influence variations in trust toward physicians. Integrating these factors into upcoming validity studies will help clarify their impact on physician trust.

## 5 Conclusion

In conclusion, this research utilized data pertaining to internal migrants in Shanghai, China, to investigate the influence of acculturation strategies on trust in physicians among this demographic. The findings revealed that the internal migrant population can be categorized into four distinct groups, each employing different acculturation strategies: integration, assimilation, separation, and marginalization. This classification extends Berry’s bidimensional acculturation strategy theory to the context of Chinese migrants. Furthermore, the study established a correlation between physician trust and varying patterns of assimilation among internal migrants, thereby providing valuable insights into the mechanisms through which internal migrants impact doctor-patient relationships in megacities like Shanghai, characterized by an indigenous population with diverse cultural backgrounds. Prospectively, in accordance with Berry’s framework for acculturation strategies, future research employing longitudinal data should integrate variables related to intergenerational and familial dynamics, alongside psychological and cultural factors. This methodology will enable the observation of evolving trends in physician trust associated with Berry’s acculturation strategy theory among internal migrants. The incorporation of these variables is anticipated to improve the validity and relevance of findings concerning physician trust within this population.

This study also has policy implications, which can provide policy recommendations for the government to promote doctor-patient relationship and improve population governance among internal migrants in megacities of China and other developing countries. By equipping migrant leaders with data-driven advocacy tools, this study fostered bottom-up approaches to address structural inequities in healthcare access. The government and community stakeholders could developing acculturation-oriented public management policies for migrant groups adopting different acculturation strategies to ensure their trust in physicians among internal migrants in China.

Firstly, to facilitate the integration of internal migrant groups employing acculturation strategies of integration and assimilation, it is essential to propose initiatives that support the maintenance of their current cultural practices. These practices may include dietary habits, clothing styles, dialect usage, media consumption, and the adaptation to local customs and traditions. For instance, initiatives designed to enhance local food culture and support experiential activities in communities with substantial migrant populations may encompass the organization of local clothing design competitions, the provision of training in local customs and languages, and the promotion of cultural exchanges with residents via social media platforms. Importantly, such training sessions were conducted for migrant community leaders to use the study’s acculturation framework to advocate for tailored health programs. This empowered them to articulate needs to policymakers. All efforts mentioned above require collaborative engagement among community health centers, local residents, and relevant governmental agencies to foster an inclusive and diverse cultural milieu. Such an environment would subsequently improve the capacity of migrants to integrate more successfully into the local community. Such measures are vital for fostering a smooth integration into local cultures, thereby enabling economic, social, and psychological assimilation within major urban centers. The processes of acculturation outlined above are likely to enhance migrants’ confidence in health care systems and build trust in local medical practitioners. This, in turn, will enable them to access essential public services and welfare provisions, promoting equitable integration into the local community. The floating indigenous population can gradually attain cultural and identity recognition, ultimately achieving full integration into urban society. Therefore, the development of public management policies aimed at promoting the integration of internal migrants from an acculturative perspective is not only critical for addressing health inequities associated with the governance of floating populations but also essential for alleviating the strained doctor-patient relationships prevalent in China. Furthermore, it is imperative for the government to consider the allocation of additional medical resources to specific demographic groups in order to enhance their utilization of outpatient services and foster greater trust in healthcare providers. Special emphasis should be placed on middle-aged and highly educated migrant populations. This could involve initiatives launched by multiple stakeholders aimed at promoting proficiency in Mandarin and the Shanghai dialect to facilitate improved communication between doctors and patients. Such measures would not only enhance the quality of medical and health services but also improve the overall experience and satisfaction with medical services, alleviate acculturation pressures, and subsequently bolster trust in physicians. Additionally, it is essential to implement strategies that elevate income levels and increase the coverage rate of the Urban Resident Basic Medical Insurance (URRMI) for both urban and rural internal migrants. These efforts would contribute to improved health service utilization and ultimately enhance self-rated health (SRH), thereby strengthening the doctor-patient relationship among internal migrants in China’s megacities.

In light of the findings presented, it is imperative that future policies focus on interventions aimed at bridging acculturation gaps. An illustrative example is Shanghai’s ‘Cultural Bridger’ program, which employs trained mediators from hospitals and migrant associations to collaboratively tackle systemic distrust. Such initiatives demonstrate how partnerships among stakeholders can effectively translate research into practices that promote equitable access to healthcare.

## Data Availability

The raw data supporting the conclusions of this article will be made available by the authors, without undue reservation.
