# Supplementary figures and images for: The influences of acculturation strategies on physician trust among internal migrants in Shanghai, China: a cross-sectional study in 2021
Source: Front Public Health. 2025 Apr 23;13:1506520. doi: 10.3389/fpubh.2025.1506520 (PMC12055845; doi:10.3389/fpubh.2025.1506520)

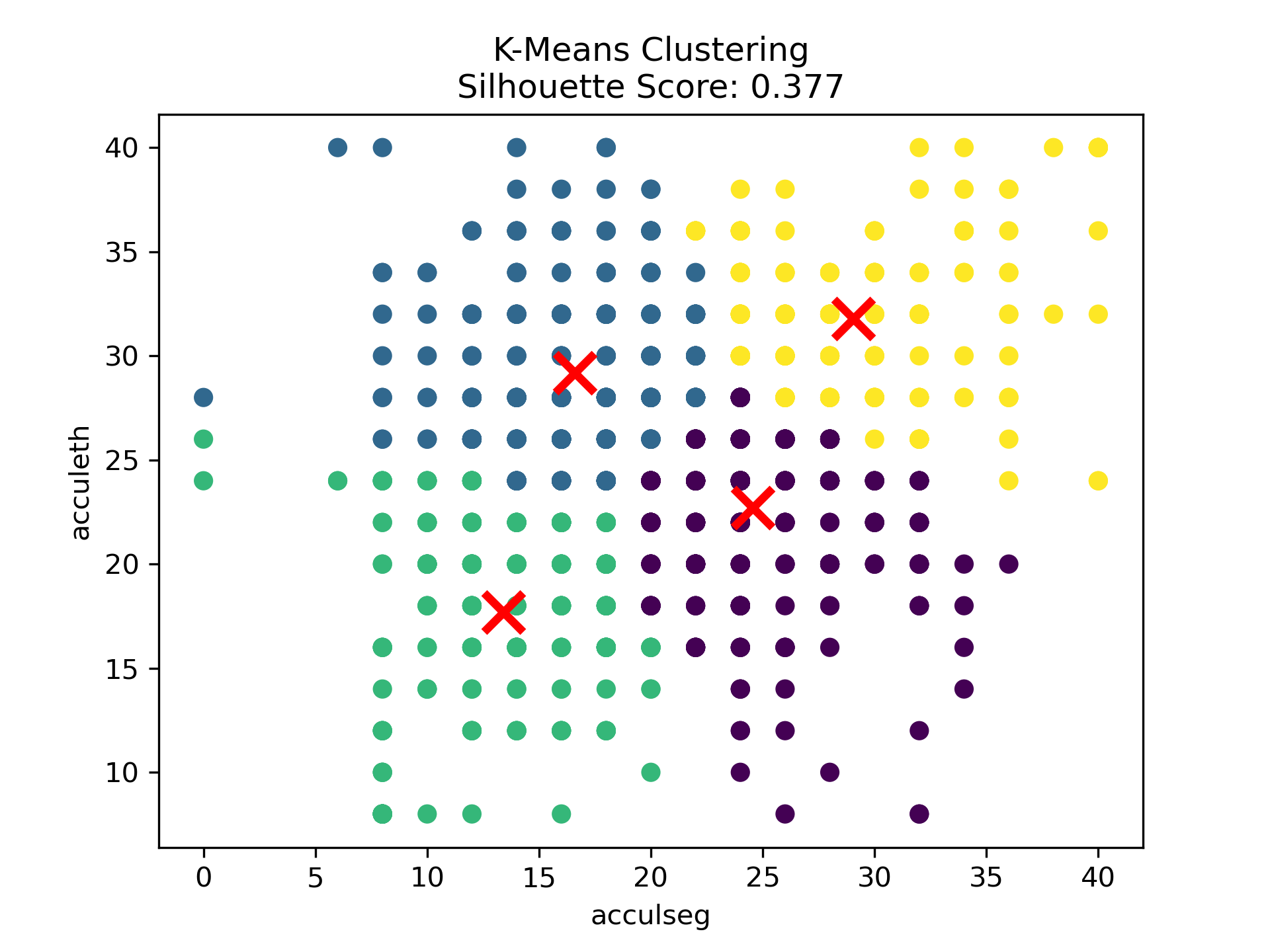

Supplement: Supplementary file 1 [file Image_1.png]
